# Research on Interface Bonding Properties of TiAlSiN/WC-Co Doped with Graphene

**DOI:** 10.3390/mi14020431

**Published:** 2023-02-11

**Authors:** Junru Yang, Yan Wang, Hao Lv, Yanping Yue, Shulei Li, Ran Zhu

**Affiliations:** College of Mechanical and Electronic Engineering, Shandong University of Science and Technology, Qingdao 266590, China

**Keywords:** TiAlSiN/WC-Co, first-principles, graphene, adhesion work, interface bonding property, electronic structure

## Abstract

Based on the first-principles method, TiAlSiN/WC-Co interface models with graphene doped into the matrix, coating, and the coating/matrix are constructed. The interface adhesion work is calculated and modeled to study the interface bonding properties from the atomic microscopic point of view. The results show that the interface bonding properties of TiAlSiN/WC-Co can be improved when the matrix is doped with the main surface of intrinsic graphene, and the interface bonding property of TiAlSiN_N_/WC-Co can be improved when the coating and coating/matrix are doped separately with the main surface of intrinsic graphene or single vacancy defective graphene. Furthermore, the model electronic structures are analyzed. The results show that there exist strong Si/Co and N/Co covalent bonds in the interfaces when the matrix is doped with the main surface of intrinsic graphene, which causes the adhesion work of TiAlSiN/WC/msGR/Co to be greater than that of TiAlSiN/WC-Co. Additionally, when the graphene is doped into the coating, in the interface of TiAlSiN/msGR/TiAlSiN_N_/WC-Co, there exist strong N/Co covalent bonds that increase the interface adhesion work. Additionally, more charge transfer and orbital hybridization exist in the coating/matrix interface doped with the main surface of intrinsic graphene or single vacancy defective graphene, which explains the essential mechanism that the adhesion work of TiAlSiN_N_/msGR/WC-Co is greater than that of TiAlSiN_N_/WC-Co, and the adhesion work of TiAlSiN_N_/svGR/WC-Co is greater than that of TiAlSiN_N_/WC-Co.

## 1. Introduction

Modern machining technologies put forward higher requirements for cutting tools [1]. Traditional WC-Co-cemented carbide tools have poor properties due to the degree of matching between the hard-phase WC and bonding phase Co in the composition. The coated cutting tool combines the coating with the tool matrix to improve its durability, thereby promoting the development of the tool [2,3].

By using the PVD method, the TiAlSiN-coated cemented carbide tool is fabricated by depositing TiAlSiN coating on the cemented carbide WC-Co matrix, which has high hardness, wear resistance, and oxidation resistance. Qiang Chen et al. [4], Yan Wu et al. [5], and Cihai Liu et al. [6] found that the TiAlSiN coating could reduce cutting force, and the coated tools had good cutting properties. However, TiAlSiN coating is prone to peeling when machining materials that are difficult to cut, and tool life is greatly reduced. Thus, the interface bonding property between the coating and matrix is crucial to obtaining a coated tool with high cutting ability. Furthermore, this has also been the emphasis and challenge for coated material research during recent years [7,8].

Graphene (GR), as a nanomaterial, has been applied in cutting-tool materials because of its special structure and properties [9,10]. Xiaorong Zhou et al. [11] added GR into WC-Co, and found that the fracture strength of the material was obviously improved. The Cu/WC/GR coating fabricated by Hatem Akbulut et al. [12] showed excellent wear resistance under a dry sliding condition. Zekai Wang et al. [13] fabricated the cutting tool with GR-doped CrAlNi coating on WC-Co, and found that the wear volume of the coating clearly decreased. Jialin Sun et al. [14] used experiments to study the influence of GR content on the toughening of WC-Co. Kui Chen et al. [15] performed XRD analysis on the WC/GR structure and found that the diffraction peak of WC was mainly provided by the (0001) surface, and GR was mainly in contact with the WC (0001) surface. Yue Liu et al. [16] studied the interface property of GR/Al composites, and found that defective graphene increased the interface bonding strength of the Al(111)/double vacancy defective GR /Al(111) interface. Yichuan Chen [17] studied the electronic structure and interface adhesion property of the defective GR (001)/Al(111) interface with the first-principles method. Shu Liu et al. [18] simulated the interface bonding property of TiAlN-coated, WC-based cemented carbide tools. Peng Wang et al. [19] observed that the TiAlN coating grew preferentially along the (111) interface. Hui Liu et al. [20] fabricated TiAlSiN coating on WC-10Co matrix, and found that the diffraction peak of TiAlSiN was mainly (111) interface. Zesheng Li et al. [21] observed the microstructure of WC doped with GR, and found that GR adhered well around WC and refined WC grains. Junru Yang et al. [22] doped WC(0001)-Co with single-layer GR and double-layer GR and found that both improved the interface bonding property of WC(0001)-Co. Qi Chen et al. [23] fabricated WC-Co-cemented carbide reinforced by GR using an electrostatic adsorption and low-pressure sintering process, and found that GR was uniformly dispersed in the WC-Co matrix. Wuli Su et al. [24] added multilayer GR as an additive into WC-6Co, and found that GR showed enhanced distribution in the Co phase, WC/WC, and WC/Co interfaces.

Currently, research on TiAlSiN-coated carbide tools mainly focuses on experimental aspects. There is less research on TiAlSiN-coated carbide tools that concerns the effect of graphene doping, from an atomic microscopic point of view, on the interface bonding property of this kind of tool coating. Therefore, using the first-principles method, we report the interface bonding properties revealed by graphene-doped TiAlSiN/WC-Co interface models in which graphene is doped into cemented carbide WC-Co matrix, TiAlSiN coating, and the coating/matrix interface. The research results are of great significance for the interface optimization design of TiAlSiN-coated carbide tools.

## 2. Model Construction Procedures

The geometry optimization of the WC, Co, GR, and TiAlSiN crystal models is performed to obtain the lowest energy and most stable lattice system.

### 2.1. Crystal Model Optimization

All calculations are based on spin-polarized density functional theory (DFT) using the CASTEP module [25,26,27]. The ultrasoft pseudo-potential (USPP) is used to describe the interactions between ionic core and valence electrons [28]. The RPBE (revised Perdew–Burke–Enzerhof) function of generalized gradient approximation (GGA) is used to manage exchange-related energy [29]. the BFGS algorithm is applied to relax the whole structure to reach the ground state where both cell parameters and fractional coordinates of atoms are optimized simultaneously [30,31]. The Brillouin zone is integrated by means of the Monkhorst–Pack scheme [32] using a grid of 7 × 7 × 7 k-point sampling for the bulk and all slabs, respectively. Plane-wave cutoff energy in all calculations is 350 eV. Additionally, the SCF convergence threshold is set as 1.0 × 10^−5^ eV/atom [33].

After setting the above parameters, the geometry optimization of WC, Co, GR and TiAlSiN crystal models is carried out. The lattice cell parameters of WC, Co, GR and TiAlSiN after geometry optimization are shown in Table 1; the optimized parameter values are consistent with experimental values in references [34,35,36].

### 2.2. Construction of Interface Models

**Figure 1:**
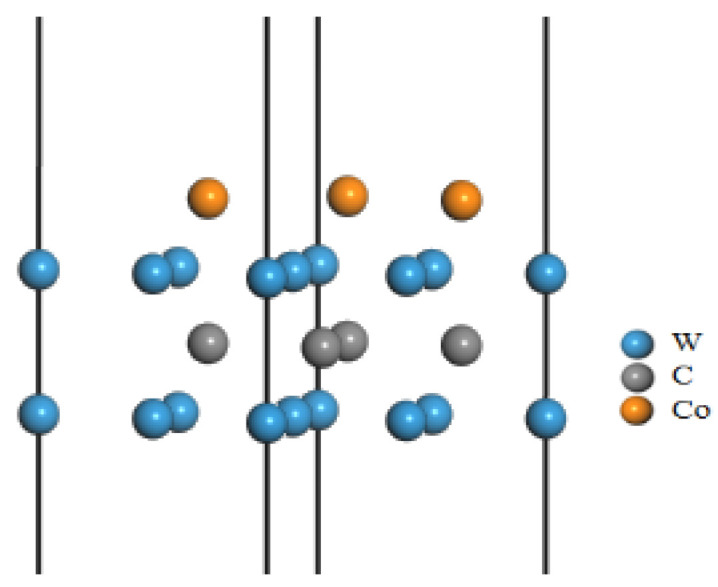
WC-Co interface model with vacuum layer.

**Figure 2:**
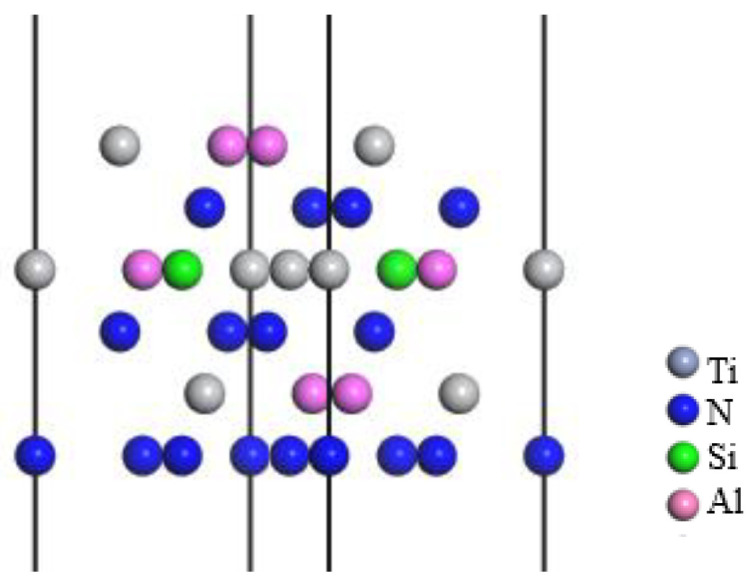
TiAlSiN interface model with vacuum layer.

The interface models are constructed by the following steps:

(1) The WC(0001) crystal face with the W atom as the terminal is the most stable [37], and the atomic distance more than three layers is too far, which has little influence on the interface. Therefore, a three layer 2 × 2 × 1 supercell WC(0001) surface is constructed, and the terminal atom is W. Some researchers observed that there were a large number of Co atoms at the grain boundary of TiAlSiN, and that Co mainly played a bonding role between WC and TiAlSiN [38]. Experiments showed that the diffraction peak of TiAlSiN was mainly the (111) plane when it was coated on WC-10Co matrix [20]. Therefore, in this study, Co atoms are added between the WC and TiAlSiN interface to build the interface model. It was found that Co can replace C atoms on the surface of WC(0001) [39], and some scholars had established the WC-Co/Diamond model by adding Co atoms at the WC interface [40]. Therefore, three C atoms on the WC (0001) surface are replaced by Co atoms, and a 10 Å vacuum layer is added to form the WC-Co interface model, as shown in Figure 1, and the interface model at this time is used to approximately represent the matrix WC-10Co.

(2) Si atoms in TiAlSiN existed in the form of replacing Ti atoms at the interface [41], and TiAlN coating grew preferentially along the interface (111) [19]. Therefore, the TiAlN crystal cell is cut along plane (111) to obtain the TiAlN (111) crystal plane, which is extended to seven layers along the vertical crystal plane direction. One Ti atom at the crystal plane is replaced by a Si atom, and a 10 Å vacuum layer is added to form the TiAlSiN interface model, as shown in Figure 2, which is used to approximately represent the TiAlSiN coating with higher hardness and better uniformity [42].

**Figure 3:**
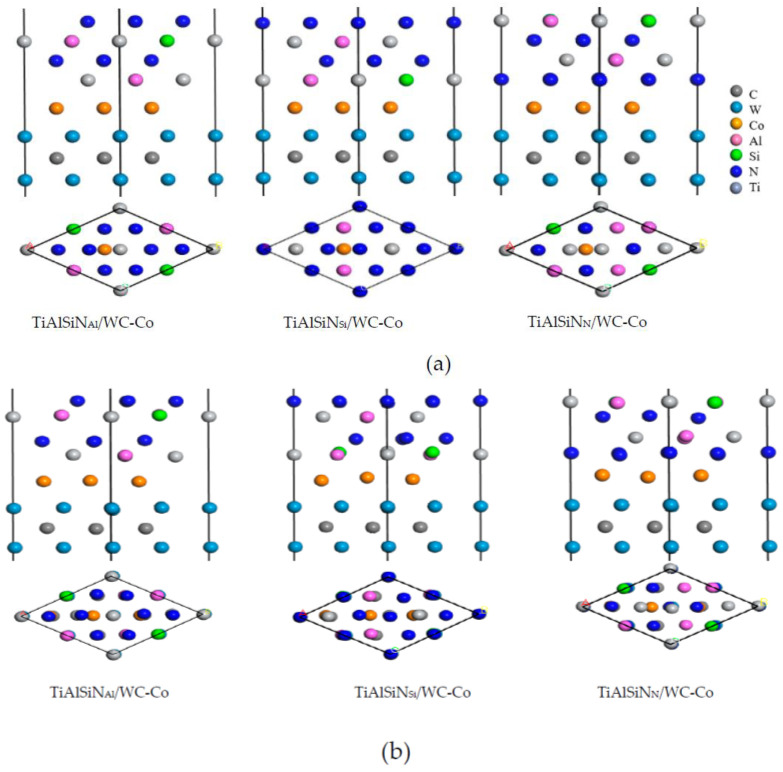
TiAlSiN/WC-Co interface models: (**a**) TiAlSiN/WC-Co interface model before optimization and (**b**) geometrically optimized TiAlSiN/WC-Co interface model.

The interface bonding strength between the TiAlSiN coating and WC-Co matrix directly affects the service life of coated carbide tools. There are three kinds of terminal atoms (Al, Si, N) on the surface of the TiAlSiN crystal. Therefore, the interface models of TiAlSiN_Al_/WC-Co, TiAlSiN_Si_/WC-Co, and TiAlSiN_N_/WC-Co without graphene doping were built, as shown in Figure 3a. Next, the three interface models were geometrically optimized. They have relaxation changes, and the internal atoms have small displacement, and their geometric dimensions have a small extension in the direction perpendicular to the interface, and little change in the direction parallel to the interface. The interface model after geometric optimization is shown in Figure 3b.

Shulei Li et al. [43] found that, among the above interface models, the model TiAlSiN_Si_/WC-Co has the strongest interface bonding property, and TiAlSiN_N_/WC-Co has the weakest interface bonding property. Therefore, based on the interface models of TiAlSiN_Si_/WC-Co and TiAlSiN_N_/WC-Co, the effect of graphene doping on the interface bonding property of TiAlSiN/WC-Co was studied.

**Figure 4:**
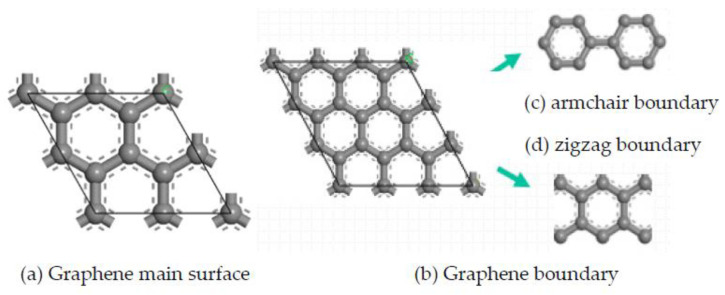
Intrinsic graphene.

**Figure 5:**
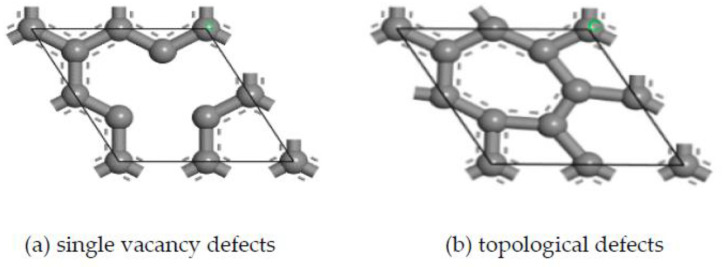
Defective graphene.

(3) Graphene mainly includes intrinsic graphene and defective graphene [44]. Intrinsic graphene, as shown in Figure 4, includes the main surface (msGR) and graphene boundary, and the graphene boundary has armchair-boundary graphene (acGR) and zigzag-boundary graphene (zzGR). Defective graphene, as shown in Figure 5, can be divided into two types of single vacancy defect graphene (svGR) and topological defect graphene (tdGR). Graphene is prone to exist between WC and Co, which accelerates the densification process of matrix grains [45]. In order to study the effect of doping graphene into the matrix on the interface bonding property of TiAlSiN/WC-Co, based on the TiAlSiN_Si_/WC-Co and TiAlSiN_N_/WC-Co interface models, the models of matrix doped with intrinsic and defective graphene, respectively, of TiAlSiN/WC-Co are constructed. The preferred orientation of graphene is (001). The most representative single-layer graphene is selected to construct a single-layer GR(001) surface, and a 10 Å vacuum layer is added to form the GR(001) interface model, which is placed between WC and Co in the WC(0001)-Co interface model. The orientation relationship is TiAlSiN(111)/WC(0001)/GR(001)/Co.

(4) Similarly, in order to study the effect of doping graphene into the coating and coating/matrix interface on the interface bonding property of TiAlSiN/WC-Co, the GR(001) interface model is placed in the TiAlSiN interface model to construct models of the coating doped with intrinsic and defective graphene. The orientation relationship is TiAlSiN(111)/GR(001)/TiAlSiN(111)/WC(0001)-Co. The GR(001) interface model is placed between the coating TiAlSiN and matrix WC-Co, and the models of coating/matrix interface doped with intrinsic and defective graphene of TiAlSiN/WC-Co are constructed. The orientation relationship is TiAlSiN(111)/GR(001)/WC(0001)-Co.

**Figure 6:**
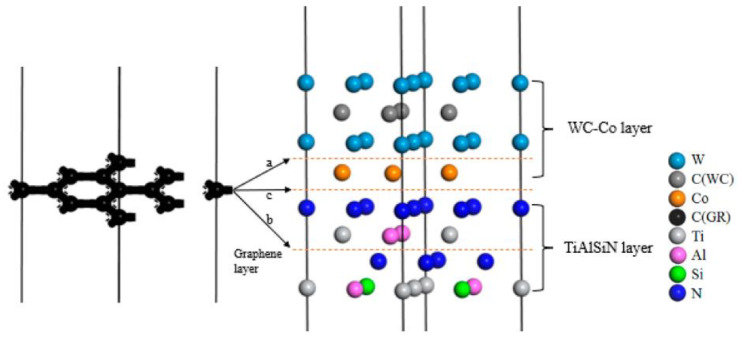
The construction process of the graphene-doped TiAlSiN/WC-Co interface model: a—represents matrix interface doped with graphene, b—represents coating interface doped with graphene, and c—represents coating/matrix interface doped with graphene.

The construction process of graphene-doped TiAlSiN/WC-Co interface model is shown in Figure 6.

**Figure 7:**
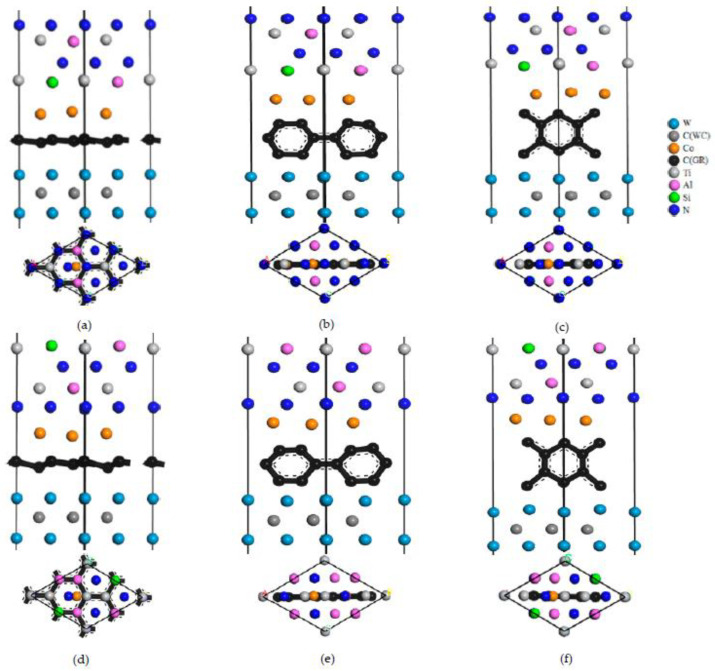
Interface models of matrix doped with intrinsic graphene: (**a**) TiAlSiN_Si_/WC/msGR/Co; (**b**) TiAlSiN_Si_/WC/acGR/Co; (**c**) TiAlSiN_Si_/WC/zzGR/Co; (**d**) TiAlSiN_N_/WC/msGR/Co; (**e**) TiAlSiN_N_/WC/acGR/Co; (**f**) TiAlSiN_N_/WC/zzGR/Co.

Specifically, based on the TiAlSiN_Si_/WC-Co and TiAlSiN_N_/WC-Co models, interface models of matrix doped with intrinsic graphene are constructed, as shown in Figure 7. In the Si terminal models, the interface models of TiAlSiN_Si_/WC/msGR/Co, TiAlSiN_Si_/WC/acGR/Co, and TiAlSiN_Si_/WC/zzGR/Co combining the matrix with the main surface of intrinsic graphene and the graphene boundary are constructed, as shown in Figure 7a–c. Similarly, for the N terminal models, the interface models of TiAlSiN_N_/WC/msGR/Co, TiAlSiN_N_/WC/acGR/Co, and TiAlSiN_N_/WC/zzGR/Co are constructed, as shown in Figure 7d–f.

Four interface models, TiAlSiN_Si_/WC/svGR/Co, TiAlSiN_Si_/WC/tdGR/Co, TiAlSiN_N_/WC/svGR/Co, and TiAlSiN_N_/WC/tdGR/Co, with defective graphene doped matrix are constructed, as shown in Figure S1.

With the same method, the interface models of coating and coating/matrix interface doped with graphene are constructed, as shown in Figures S2–S5.

## 3. Results and Discussion

The adhesion work can characterize the bonding strength of the interface structure. The greater the adhesion work, the stronger the interface bonding property and the more stable the interface structure. Furthermore, the charge-density difference and the density of states are analyzed by calculating the electronic structures of the model interface atoms, revealing the essence of interface bonding from the perspective of charge transfer and bonding mode.

### 3.1. Adhesion Work

The interface *α/β* adhesion work (Wad) calculation formula is [46]:

(1) $$W_{ad}=\frac{E_{\alpha }+E_{\beta }-E_{\alpha /\beta }}{A_{\alpha /\beta }}$$

where *W_ad_* is the adhesion work, J/m^2^; *E_α_* is the total energy of surface *α*, eV; *E_β_* is the total energy of surface *β*, eV; *E_α/β_* is the total energy of the interface system, eV; and the total interface area is given by *A*, Å^2^.

The calculated interface adhesion works of matrix doped with intrinsic graphene are shown in Table 2. When the matrix is doped with the main surface of intrinsic graphene, the interface adhesion work of TiAlSiN_Si_/WC/msGR/Co is larger than that of TiAlSiN_Si_/WC-Co without graphene doping, and the interface adhesion work of TiAlSiN_N_/WC/msGR/Co is larger than that of TiAlSiN_N_/WC-Co, which indicates that the combination of the matrix and the main surface of intrinsic graphene improves the interface bonding property of TiAlSiN/WC-Co. When the matrix is doped with the graphene boundary, the adhesion works of TiAlSiN_Si_/Co interface and TiAlSiN_N_/Co interface are less than those without doping, which indicates that the combination of matrix and graphene boundary reduces the interface bonding property of TiAlSiN/WC-Co.

The calculated interface adhesion works of matrix doped with defective graphene are shown in Table 3. When the matrix is doped with svGR and tdGR, the adhesion works of TiAlSiN_Si_/Co and TiAlSiN_N_/Co are less than those without doping, respectively, which indicates that the combination of matrix and defective graphene reduces the interface bonding property of TiAlSiN/WC-Co.

By comparing adhesion works of Si and N terminal models of matrix doped with graphene, given in Table 2 and Table 3, it can be concluded that when the matrix is doped with graphene, doping msGR can increase interface adhesion works of Si and N terminal models. When matrix is doped with other forms of graphene, interface adhesion works of Si and N terminal models decrease in varying degrees, and adhesion works of Si terminal models clearly decrease. In the N terminal models, when matrix is doped with tdGR, the interface adhesion work is the least and the interface bonding property is the worst. In the Si terminal models, the interface adhesion work is the least and the interface bonding property is the worst when matrix is doped with acGR, and there is a situation that the adhesion work of the Si terminal model is less than that of the N terminal model at this time, and adhesion works of Si terminal models with the matrix doped with other forms of graphene are larger than those of N terminal models. It can be seen that the interface bonding property decreases most obviously when the Si terminal model is doped with acGR.

The calculated interface adhesion works of coating doped with intrinsic graphene are shown in Table 4. When the coating is doped with the main surface of intrinsic graphene, the adhesion work of the TiAlSiN_Si_/Co interface of TiAlSiN/msGR/TiAlSiN_Si_/WC-Co is less than that of the TiAlSiN_Si_/Co interface of TiAlSiN_Si_/WC-Co without graphene doping, and the interface bonding property of TiAlSiN/msGR/TiAlSiN_Si_/WC-Co decreases.

The adhesion works of the TiAlSiN_N_/Co interface of TiAlSiN/msGR/TiAlSiN_N_/WC-Co are larger than that of the TiAlSiN_N_/Co interface of TiAlSiN_N_/WC-Co without graphene doping, and the interface bonding property of TiAlSiN/msGR/TiAlSiN_N_/WC-Co increases. The results indicate that the coating is doped with the main surface intrinsic graphene in the N terminal model, and the interface bonding property of TiAlSiN/msGR/TiAlSiN_N_/WC-Co increases. When the coating is doped with the graphene boundary, the adhesion works of TiAlSiN_Si_/Co and TiAlSiN_N_/Co are less than those without doping, which indicates that the combination of coating and graphene boundary reduces the interface bonding property of TiAlSiN/WC-Co.

The calculated interface adhesion works of coating doped with defective graphene are shown in Table 5. The adhesion work results show that only when the coating in the N terminal model of TiAlSiN_N_/WC-Co is doped with svGR, the adhesion work of each interface increases compared with that of TiAlSiN_N_/WC-Co without doping, and the interface bonding property increases. When the coating is doped with svGR, in the Si terminal model of TiAlSiN_Si_/WC-Co and different terminal models of doping tdGR, there are interfaces whose adhesion works decrease, and the interface bonding property decreases.

By comparing adhesion works of Si and N terminal models doped with graphene in coating, given in Table 4 and Table 5, it can be concluded that when coating is doped with graphene, the interface adhesion works of Si terminal models decrease in varying degrees, and the interface bonding property decreases. The interface adhesion work is the least and the interface bonding property is the worst when coating is doped with zzGR. In the N terminal models, when the coating is doped with msGR and svGR, the interface adhesion works increase in varying degrees, and the interface bonding property improves. However, the interface adhesion works decrease when coating is doped with acGR, zzGR, and tdGR, and the interface adhesion work is the least when the coating is doped with acGR, and the bonding property is the worst. Compared with the coating without graphene, when the coating is doped with graphene, the adhesion works of Si terminal models are less than those of N terminal models. It can be seen that the interface bonding property decreases most obviously when Si terminal models are doped with graphene.

The calculated interface adhesion works of the coating/matrix interface doped with intrinsic graphene are shown in Table 6. It can be seen that when the coating/matrix interface is doped with the main surface of intrinsic graphene, the interface adhesion work of TiAlSiN_Si_/msGR/WC-Co of the Si terminal model is less than that of the TiAlSiN_Si_/WC-Co interface without doping graphene, and the interface bonding property of TiAlSiN_Si_/msGR/WC-Co decreases. The interface adhesion work of TiAlSiN_N_/msGR/WC-Co of the N terminal model is larger than that of the TiAlSiN_N_/WC-Co interface, and the interface bonding property of TiAlSiN_N_/msGR/WC-Co increases. When the coating/matrix interface is doped with the graphene boundary, in different terminal models, there are interfaces whose interface adhesion works are less than that of the TiAlSiN/WC-Co interface. It indicates that when the coating/matrix interface is doped with an intrinsic graphene boundary, the interface bonding property of TiAlSiN/WC-Co decreases.

The calculated interface adhesion works of the coating/matrix interface doped with defective graphene are shown in Table 7. The adhesion work results show that only when the coating/matrix interface in TiAlSiN_N_/WC-Co is doped with svGR does the adhesion work of each interface increase compared with that of TiAlSiN_N_/WC-Co without doping, and the interface bonding property increases. In TiAlSiN_Si_/svGR/WC-Co, TiAlSiN_Si_/tdGR/WC-Co, and TiAlSiN_Si_/tdGR/WC-Co, there are interfaces whose interface adhesion works decrease, and the interface bonding property decreases.

By comparing adhesion works of Si and N terminal models doped with graphene in coating/matrix interface, given in Table 6 and Table 7, it can be concluded that when the coating/matrix interface is doped with graphene, the interface adhesion works of Si terminal models decrease in varying degrees, and the interface bonding property decreases. The interface adhesion work is the least and the interface bonding property is the worst when the coating/matrix interface is doped with msGR. In the N terminal models, when the coating/matrix interface is doped with msGR and svGR, the interface adhesion works increase in varying degrees, and the interface bonding property improves, and the interface adhesion work is the largest when the coating/matrix interface is doped with msGR. However, the interface adhesion work decreases when the coating/matrix interface is doped with acGR, zzGR, and tdGR, and the interface adhesion work is the least when the coating/matrix interface is doped with acGR, and the bonding property is the worst. When the coating/matrix interface is doped with acGR, svGR, and tdGR, the adhesion work of the Si terminal model is larger than that of N terminal model. When the coating/matrix interface is doped with msGR and zzGR, there is a situation that the adhesion work of the Si terminal model is less than the N terminal model. It can be seen that the interface bonding property decreases most obviously when Si terminal models are doped with grapheme.

**Figure 8:**
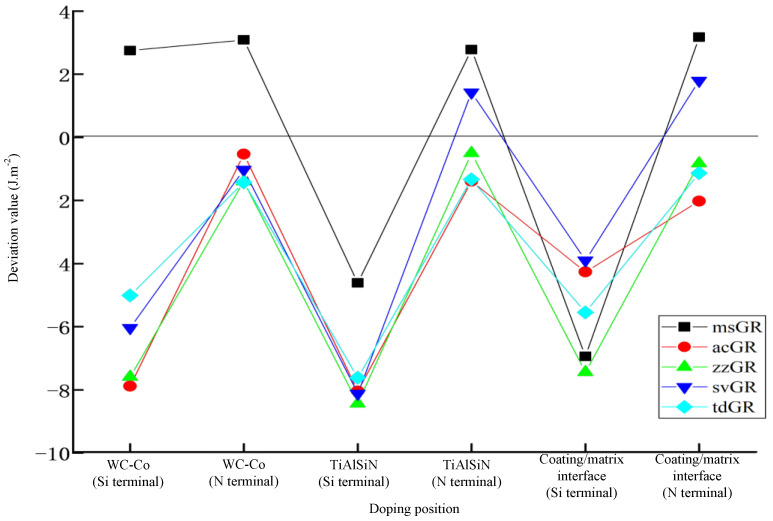
Deviation between adhesion work of graphene-doped TiAlSiN/WC-Co and that of TiAlSiN/WC-Co without graphene doping.

After calculation, the adhesion works of the graphene-doped TiAlSiN/WC-Co interface model were summarized, as shown in Table 8. The influence of graphene doping on the interface bonding property of TiAlSiN/WC-Co is analyzed. Figure 8 shows the deviation between the adhesion work of graphene-doped TiAlSiN/WC-Co and that of TiAlSiN/WC-Co without graphene doping. The results show that in the N terminal model, the differences between the adhesion work of TiAlSiN/WC-Co doped with graphene in WC-Co or TiAlSiN and that of TiAlSiN/WC-Co without graphene doping are small. In the Si terminal model, the differences between the adhesion work of TiAlSiN/WC-Co doped with graphene in WC-Co and that of TiAlSiN/WC-Co without graphene doping are significant.

It can be seen from Table 8 that, from the doping position, when the matrix is doped with graphene, msGR improves the interface bonding properties of both Si and N terminal models, while other forms of graphene doping cannot improve their interface bonding property. When the coating is doped with graphene, msGR and svGR improve the interface bonding properties of TiAlSiN_N_/WC-Co, while other forms of graphene doping cannot improve the interface bonding property of TiAlSiN/WC-Co. When the coating/matrix interface is doped with graphene, msGR and svGR improve the interface bonding properties of TiAlSiN_N_/WC-Co, while other forms of graphene doping cannot improve the interface bonding property of TiAlSiN/WC-Co.

From the perspective of different forms of graphene doping, it is found that when msGR is doped into the matrix of Si terminal models, the interface bonding property of TiAlSiN_Si_/WC-Co increases; when msGR is doped into the matrix, coating, and the coating/matrix interface of N terminal models, the interface bonding property of TiAlSiN_N_/WC-Co increases, but acGR, zzGR, and tdGR cannot improve the interface bonding property of TiAlSiN/WC-Co.

### 3.2. Electronic Structure

(1) Charge-density difference

**Figure 9:**
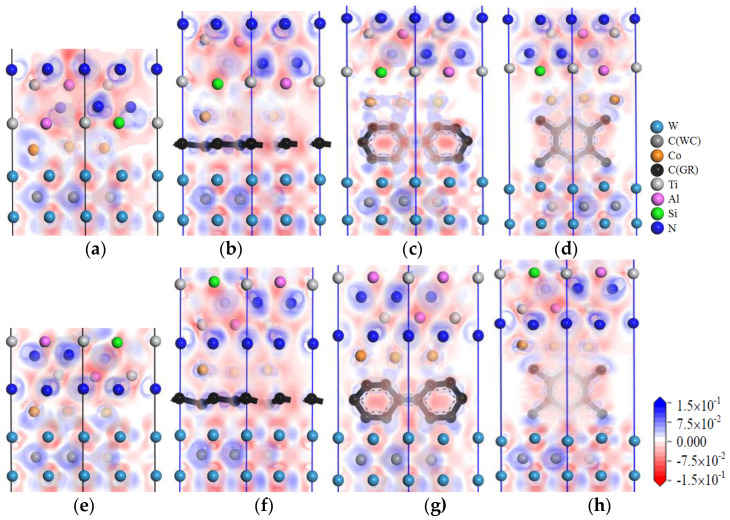
Charge-density difference image of the interface models with intrinsic graphene doping in the matrix: (**a**) TiAlSiN_Si_/WC-Co; (**b**) TiAlSiN_Si_/WC/msGR/Co; (**c**) TiAlSiN_Si_/WC/acGR/Co; (**d**) TiAlSiN_Si_/WC/zzGR/Co; (**e**) TiAlSiN_N_/WC-Co; (**f**) TiAlSiN_N_/WC/msGR/Co; (**g**) TiAlSiN_N_/WC/acGR/Co; (**h**) TiAlSiN_N_/WC/zzGR/Co.

**Figure 10:**
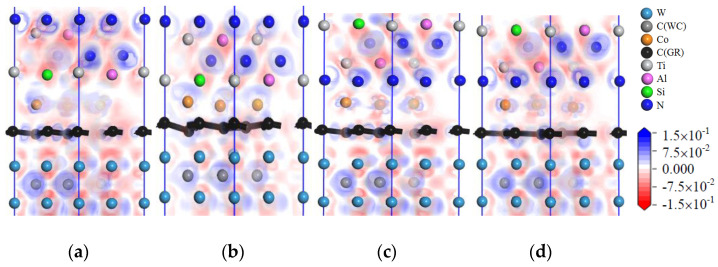
Charge-density difference image of the interface models with defective graphene doping in the matrix: (**a**) TiAlSiN_Si_/WC/svGR/Co; (**b**) TiAlSiN_Si_/WC/tdGR/Co; (**c**) TiAlSiN_N_/WC/svGR/Co; (**d**) TiAlSiN_N_/WC/tdGR/Co.

**Figure 11:**
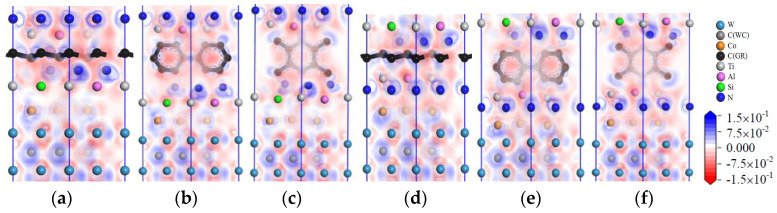
Charge-density difference image of the interface models with intrinsic graphene doping in the coating: (**a**) TiAlSiN/msGR/TiAlSiN_Si_/WC-Co; (**b**) TiAlSiN/acGR/TiAlSiN_Si_/WC-Co; (**c**) TiAlSiN/zzGR/TiAlSiN_Si_/WC-Co; (**d**) TiAlSiN/msGR/TiAlSiN_N_/WC-Co; (**e**) TiAlSiN/acGR/TiAlSiN_N_/WC-Co; (**f**) TiAlSiN/zzGR/TiAlSiN_N_/WC-Co.

**Figure 12:**
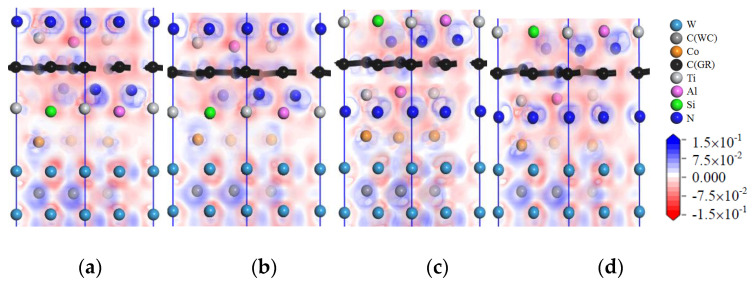
Charge-density difference of interface models that the coating is doped with defective graphene: (**a**) TiAlSiN/svGR/TiAlSiN_Si_/WC-Co; (**b**) TiAlSiN/tdGR/TiAlSiN_Si_/WC-Co; (**c**) TiAlSiN/svGR/TiAlSiN_N_/WC-Co; (**d**) TiAlSiN/tdGR/TiAlSiN_N_/WC-Co.

**Figure 13:**
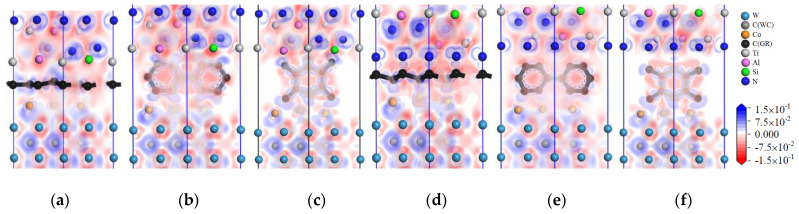
Charge-density difference image of the interface models with intrinsic graphene doping in the coating/matrix interface: (**a**) TiAlSiN_Si_/msGR/WC-Co; (**b**) TiAlSiN_Si_/acGR/WC-Co; (**c**) TiAlSiN_Si_/zzGR/WC-Co; (**d**) TiAlSiN_N_/msGR/WC-Co; (**e**) TiAlSiN_N_/acGR/WC-Co; (**f**) TiAlSiN_N_/zzGR/WC-Co.

**Figure 14:**
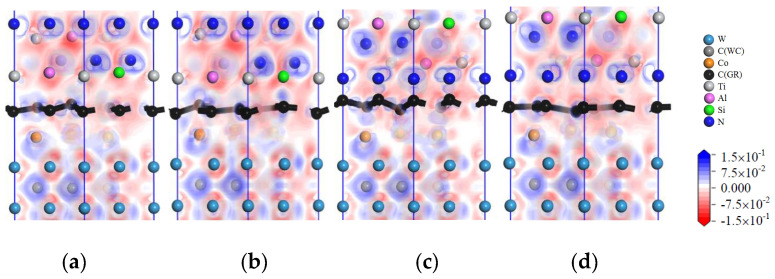
Charge-density difference image of interface models with defective graphene doping in the coating/matrix interface: (**a**) TiAlSiN_Si_/svGR/WC-Co; (**b**) TiAlSiN_Si_/tdGR/WC-Co; (**c**) TiAlSiN_N_/svGR/WC-Co; (**d**) TiAlSiN_N_/tdGR/WC-Co.

The charge-density distribution in atoms can be analyzed by the charge-density difference image. The charge-density difference of TiAlSiN_Si_/WC-Co and TiAlSiN_N_/WC-Co interface models is calculated, and the charge-density difference images are shown in Figure 9, Figure 10, Figure 11, Figure 12, Figure 13 and Figure 14. In the figures, the red, blue, and white areas indicate the decreased, increased, and approximately unchanged charge density, respectively.

Figure 9 shows the charge-density difference images of the interface models with intrinsic graphene doping in the matrix. In Figure 9a, at the TiAlSiN_Si_/WC-Co interface, there is charge accumulation between Si atoms and Co atoms, that is, covalent bonds exist; when the matrix is doped with msGR, this introduces msGR/Co and /WC/msGR into the interfaces. As shown in Figure 9b, there is obvious electron transfer between Si and Co atoms at the interface of TiAlSiN_Si_/WC/msGR/Co, and the electron cloud near the Co atom moves toward the Si atom, which enhances the attraction of Si to the Co atom and forms a strong Si/Co covalent bond. As shown in Figure 9e, the charge accumulation between N and Co atoms at the TiAlSiN_N_/WC-Co interface indicates the existence of covalent bonds. As shown in Figure 9f, at the interface of TiAlSiN_N_/WC/msGR/Co, N atoms are in the electron-enriched state, while Co atoms are in the electron-absent state, and there is attraction between N and Co atoms. When the matrix is combined with msGR, the newly introduced interface is stable and the original interface is well combined. In the four interface models, in which the matrix is doped with the graphene boundary, as shown in Figure 9c,d,g,h, there is a certain bonding force between C and Co and W atoms, while there is a charge gap between TiAlSiN and Co atoms, and the bonding force between atoms is weak, which leads to a decrease in the interface bonding property of TiAlSiN/WC-Co. When the matrix is doped with acGR, compared with the N terminal model, the Si terminal model has more charge blank areas at the interface, and the forces between Si atoms and Co and C atoms are small, and the interface bonding property decreases. The results are consistent with those shown in Table 2 and Table 3, that the adhesion work of the Si terminal model is less than that of the N terminal model.

Figure 10 shows images of the calculated charge-density difference of four interface models in which the matrix is doped with defective graphene. It is found that when the matrix is doped with defective graphene, the combination of matrix and defective graphene changes the charge distribution at the interface. There exists electron accumulation between W and C atoms of defective graphene, that is, a covalent bond exists. There are blank areas in the electron clouds between TiAlSiN and Co atoms; the charge density between atoms cannot overlap well, and the bonding between Co and Al and Si and N atoms is weak. There are also blank areas in the charge density between Co and defective graphene, and the bonding between Co and C atoms is weak, which reduces the interface bonding properties of TiAlSiN_Si_/WC/svGR/Co, TiAlSiN_Si_/WC/tdGR/Co, TiAlSiN_N_/WC/svGR/Co, and TiAlSiN_N_/WC/tdGR/Co.

Figure 11 shows images of the charge-density difference of the interface models with intrinsic graphene doping in the coating. When the coating is doped with msGR, in the model of TiAlSiN/msGR/TiAlSiN_Si_/WC-Co, shown in Figure 11a, there are electronic blank areas between Al, Si, and Co atoms, and the force between the atoms is relatively small. In the model of TiAlSiN/msGR/TiAlSiN_N_/WC-Co, shown in Figure 11d, there is electronic overlap between N and Co, and the bonding force between atoms is relatively large. There are many electrons overlapping between atoms in the other interfaces, and the interface bonding property is strengthened. When the coating is doped with an intrinsic graphene boundary, the charge between atoms at the interface between TiAlSiN and Co is thin, and the interatomic force is weakened, resulting in the decrease in the bonding property of the TiAlSiN/WC-Co interface. Compared with the coating without graphene doping, when the coating is doped with graphene, there are more electron blank areas between atoms at the interface of the Si terminal model, and the interatomic force is small. It can be seen that the interface bonding property decreases most obviously when the Si terminal model is doped with graphene, which is consistent with the results shown in Table 4 and Table 5, which indicate that the adhesion work of the Si terminal model is less than that of the N terminal model when the coating is doped with msGR and zzGR.

Figure 12 shows the image of the charge-density difference of interface models in which the coating is doped with defective graphene. In Figure 12a, there are charge blank areas between Al, Si, and Co in TiAlSiN/svGR/TiAlSiN_Si_/WC-Co, and the interatomic force is weak. As shown in Figure 12b, there are charge blank areas between Al, Si, and Co in TiAlSiN/tdGR/TiAlSiN_Si_/WC-Co. In Figure 12c, there are many electrons overlapping between C and N, Al atoms, and between Co and N atoms in TiAlSiN/svGR/TiAlSiN_N_/WC-Co, and the interatomic force is strong. In Figure 12d, the charge density between C and Al atoms and between Co and N atoms is thin, and the force between atoms is weak. Therefore, only the atomic charges overlap more at the interface of TiAlSiN/svGR/TiAlSiN_N_/WC-Co, and the interatomic force is strong. In other cases, the doping of defective graphene weakens the bonding force between atoms at the interface.

Figure 13 shows images of the charge-density difference of the interface models with intrinsic graphene doping in the coating/matrix interface. When the coating/matrix interface is doped with msGR, at the TiAlSiN_Si_/msGR/WC-Co interface, there are charges transferred between C atoms of msGR and Al atom, and there are shared charges between C and Si atoms. The charge density between Co and C atoms is thin, and the interatomic force is small. At the interface of TiAlSiN_N_/msGR/WC-Co, there are obvious shared charges between C atoms of msGR and N, Co atoms, and the interatomic force is strong. When the coating/matrix interface is doped with the graphene boundary, the combination of coating/matrix interface and graphene boundary makes the charge between atoms thin. The interatomic force decreases, and the interface bonding property decreases. Compared with the N terminal model, when the coating/matrix interface is doped with msGR and zzGR, there are more electronic blank areas at the interface of the Si terminal model, and the interatomic force is small, and the interface bonding property decreases. It can be seen that the interface bonding property decreases most obviously when the Si terminal model is doped with graphene, and the results are consistent with those given in Table 6 and Table 7.

Figure 14 shows images of the charge-density difference of interface models with defective graphene doping in the coating/matrix interface. As shown in Figure 14a, in the model of TiAlSiN_Si_/svGR/WC-Co, the charge density between Al, Si, and Co atoms is thin, there are charge blank areas between C and Co atoms, and the interatomic force is weak. In Figure 14b, in the model of TiAlSiN_Si_/tdGR/WC-Co, the charge density between Al, Si, and C atoms is thin. The charge density between Co and C atoms is thin and there are blank areas. In Figure 14c, in the model of TiAlSiN_N_/svGR/WC-Co, the electrons between C and N atoms and between C and Co atoms all overlap more, and the bonding force is strong. In Figure 14d, in the model of TiAlSiN_N_/tdGR/WC-Co, the charge density between C and Co atoms is thin, and the interatomic force is weak. Summarizing, only the atomic charges at the interface of TiAlSiN_N_/svGR/WC-Co overlap more, and the bonding force between atoms is strong. In other cases, the defective graphene doping weakens the bonding force between atoms at the interface, and the interface bonding property decreases.

(2) Density of states (DOS)

Analyzing the density of states can explore the bonding situation from the perspective of electron orbital hybridization.

**Figure 15:**
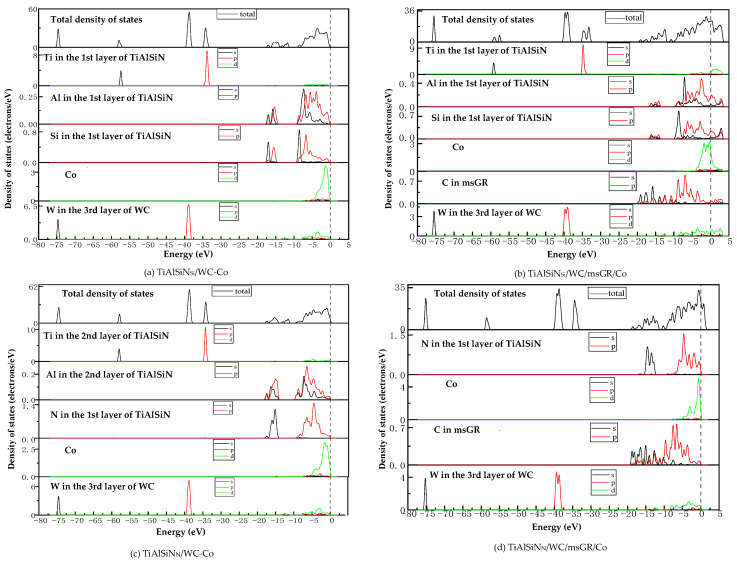
TDOS and PDOS of the interface models without doping GR and with matrix doped with msGR.

Figure 15a,c are the images of the total density of states (TDOS) and the partial density of states (PDOS) of the interface models without doping graphene. At the Fermi level, the TDOS of the interface models is not zero, indicating a metallic character for the interface. As shown in Figure 15a, the remarkable DOS feature of TiAlSiN_Si_/WC-Co is that its peak is mainly contributed by p orbital electrons of Ti atoms and W atoms, and the interaction between Si and Co at the interface is mainly the hybridization of p electrons of Si and d electrons of Co, forming Si/Co covalent bonds. Figure 15b,d are the images of TDOS and PDOS of the interface models with the matrix doped with msGR. In Figure 15b, the density of states of Al, Si, and Co atoms of TiAlSiN_Si_/WC/msGR/Co overlaps in the range −5~5 eV, and the orbital hybridization is generated by Al-s, Si-s, and Co-d, forming Al/Co bonds and Si/Co bonds. Compared with Figure 15a without graphene doping, the covalent effect of the Si/Co bond is stronger, which explains the essential mechanism that the adhesion work value of TiAlSiN_Si_/WC/msGR/Co is higher than that of TiAlSiN_Si_/WC-Co.

A shown in Figure 15c, the remarkable feature of the density of states of TiAlSiN_N_/WC-Co is that its peak is mainly contributed by p orbital electrons of Ti and W atoms, and the interaction between N and Co at the interface is mainly the hybridization of p electrons of N and d electrons of Co, forming N/Co covalent bonds. In Figure 15d, at the TiAlSiN_N_/WC/msGR/Co interface, the density of states of N and Co atoms overlaps on the left side of Fermi level, and N-s and Co-d produce orbital hybridization, forming N/Co bonds. On the left side of Fermi level, the density of states of the C atom, Co atom, and W atom is orbitally hybridized by C-p with Co-d and W-d, forming C/Co and C/W bonds. Compared with Figure 15c, the electronic orbital hybrid region of N/Co, C/Co, and C/W bonds changes little, and the peak change in density of states of interface atomic interaction is small, and the bonding strength is stable. Therefore, the adhesion work of TiAlSiN_N_/WC/msGR/Co is greater than that of TiAlSiN_N_/WC-Co.

**Figure 16:**
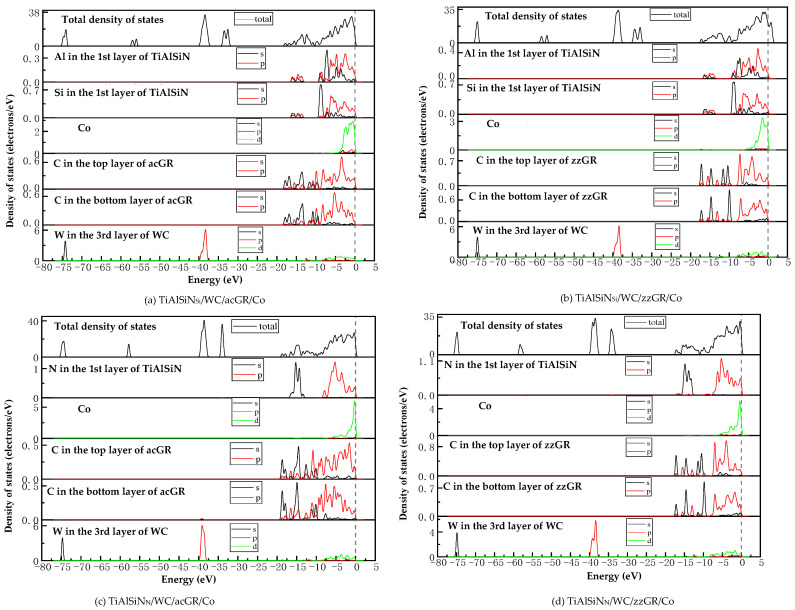
TDOS and PDOS of the interface models with the matrix doped with an intrinsic graphene boundary.

Figure 16 shows the images of TDOS and PDOS of the interface models with the matrix doped with an intrinsic graphene boundary. Compared with undoped graphene, when the matrix is doped with an intrinsic graphene boundary, C-p of C atoms at the graphene boundary, Co-d and W-d produce orbital hybridization, forming C/Co and C/W bonds. The degree of electronic orbital hybridization of Al, Si, N, and Co atoms at the TiAlSiN interface is weak, and the bonding effect is weak, which leads to the decrease in the bonding property of the TiAlSiN/WC-Co interface. Therefore, the adhesion work of the TiAlSiN/Co interface of different terminal models with matrix doped with an intrinsic graphene boundary is less than that of TiAlSiN/Co interface without graphene doping.

**Figure 17:**
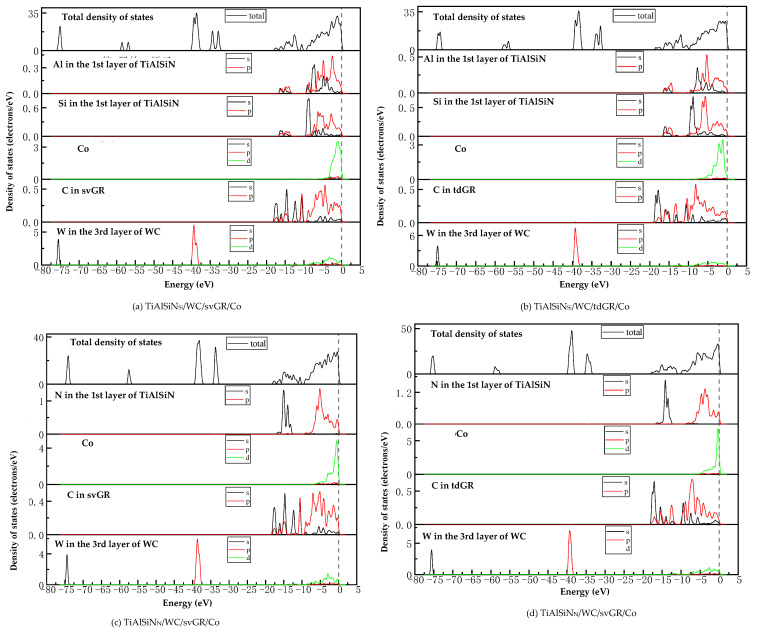
TDOS and PDOS of the interface models with the matrix doped with defective graphene.

Figure 17 shows the images of TDOS and PDOS of the interface models with the matrix doped with defective graphene. As shown in Figure 17a, C-p of C atom of svGR, Co-d, and W-d produce orbital hybridization, forming C/Co and C/W bonds. The density of states of Al, Si, and Co atoms overlaps in the range −5~0 eV. The density of states of the Si atom is low, and the degree of hybridization with Co-d orbitals is weak. In Figure 17b, the density of states of Al, Si, and Co atoms in the overlapping range in TiAlSiN_Si_/WC/tdGR/Co is low, below 0.2 eV, and the degree of orbital hybridization of Al-p, Si-p, and Co-d is weak. In Figure 17c, the density of states region of Co atoms in TiAlSiN_N_/WC/svGR/Co is narrow, resulting in low orbital hybridization of Co-d with C-p and N-p, and weak interatomic bonding force. In Figure 17d, the orbital hybridization degree of N-p and Co-d in TiAlSiN_N_/WC/tdGR/Co is low, and the interatomic bonding force is weak. This explains the essential mechanism that the interface adhesion work of TiAlSiN/WC-Co decreases when its matrix is doped with defective graphene.

**Figure 18:**
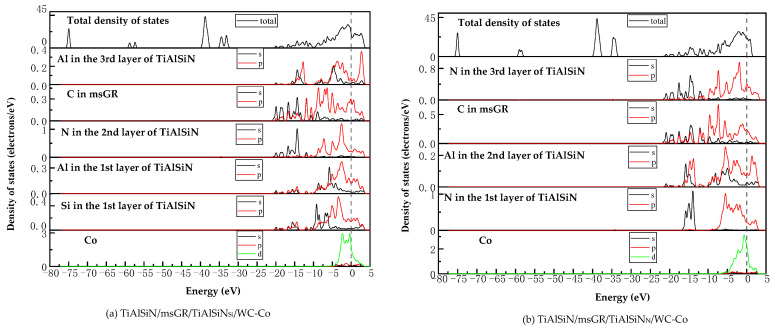
TDOS and PDOS of the interface models with the coating doped with msGR.

Figure 18 shows the images of TDOS and PDOS of the interface models with the coating doped with msGR. As shown in Figure 18a, at the interface of TiAlSiN/msGR/TiAlSiN_Si_/WC-Co, the charges between Al, Si, and Co atoms are rearranged, and at Fermi level, Al, Si, and Co atoms at the interface produce orbital hybridization, forming covalent bonds. Compared with Figure 15a without graphene doping, the covalent effect is weak, which explains the essential mechanism that the adhesion work value of TiAlSiN/msGR/TiAlSiN_Si_/WC-Co is higher than that of TiAlSiN_Si_/WC-Co. In Figure 18b, in the model of TiAlSiN/msGR/TiAlSiN_N_/WC-Co, N-s and C-s produce orbital hybridization in the range −22~15 eV, and N-p and C-p produce orbital hybridization in the range −10~3 eV, forming N/C bonds. Al-s, Al-p and C-s, and C-p produce orbital hybridization in the range −17~3 eV, forming Al/C bonds. N-p and Co-d produce orbital hybridization in the range −5~3 eV, forming N/Co bonds. The density of atomic states of forming chemical bonds and orbital hybridization degree is high, which explains the essential mechanism that the adhesion work of TiAlSiN/msGR/TiAlSiN_N_/WC-Co is greater than that of TiAlSiN_N_/WC-Co.

**Figure 19:**
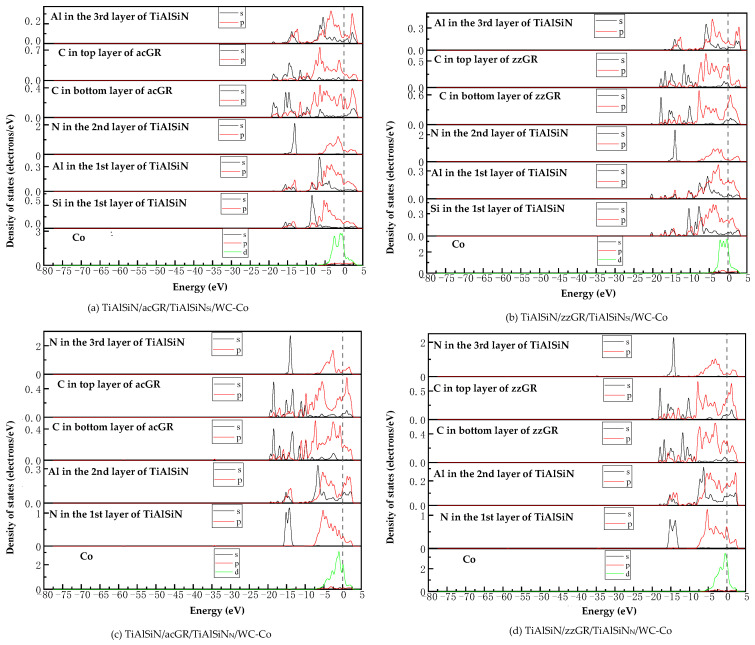
PDOS of the interface models with the coating doped with an intrinsic graphene boundary.

Figure 19 shows the images of PDOS of the interface models with the coating doped with an intrinsic graphene boundary. As shown in Figure 19a, the density of states of N-p and C-p in the model of TiAlSiN/acGR/TiAlSiN_Si_/WC-Co overlaps in the range −6~3 eV, the N-p density of states is below 0.1 eV, and the energy is low, and the N/Co bond is weak. The density of states of Al and Si atoms is low, below 0.3 eV, and the degree of hybridization with Co-d orbitals is weak. In Figure 19b, the density of states of Al-p and Si-p is low, less than 0.3 eV, and the degree of hybridization with Co-d orbitals is weak. In Figure 19c, at the TiAlSiN/acGR/TiAlSiN_N_/WC-Co interface, the charges between N and Co atoms are rearranged, and at the Fermi level, N and Co atoms at the interface produce orbital hybridization, and the degree of orbital hybridization is low, forming weak covalent bonds. In Figure 19d, at the interface of TiAlSiN/zzGR/TiAlSiN_N_/WC-Co, Co and N atoms produce orbital hybridization, the hybridization area is small, and the energy is low, and the Co/N bond is weak. This explains the essential mechanism for the decrease in adhesion work at the TiAlSiN/WC-Co interface when the coating is doped with an intrinsic graphene boundary.

**Figure 20:**
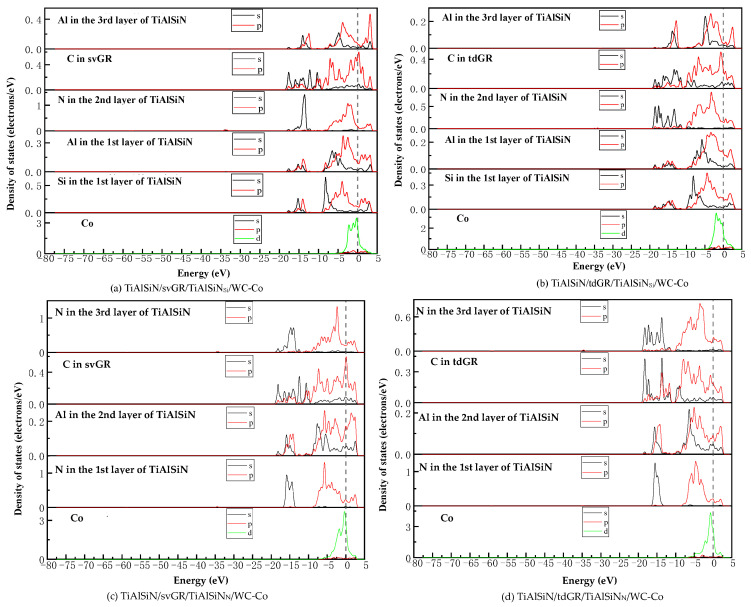
PDOS of the interface models with the coating doped with defective graphene.

Figure 20 shows the images PDOS of the interface models with the coating doped with defective graphene. As shown in Figure 20a, the Co-d, Al-p, and Si-p in the model of TiAlSiN/svGR/TiAlSiN_Si_/WC-Co produce orbital hybridization in the range −4~3 eV, and the degree of hybridization is low, forming weak Co/Si bonds. In Figure 20b, the density of states of Al, Si, and Co atoms in TiAlSiN/tdGR/TiAlSiN_Si_/WC-Co overlaps in the range −4~3 eV, where the density of states of Co is low, and the degree of orbital hybridization of Al-p, Si-p, and Co-d is weak. In Figure 20c, C-p and N-p, Al-p, Co-d, and N-p orbital hybridization regions in TiAlSiN/svGR/TiAlSiN_N_/WC-Co are wide, and the C/N, C/Al, and Co/N bonds are strong. In Figure 20d, the degree of orbital hybridization of C-p and N-p, and Al-p in TiAlSiN/tdGR/TiAlSiN_N_/WC-Co is high, while the N-p density of states is low, and the degree of orbital hybridization of Co-d and N-p is weak, and bonding force is weak. Therefore, the interface adhesion work of TiAlSiN/svGR/TiAlSiN_N_/WC-Co is greater than that of TiAlSiN_N_/WC-Co without graphene doping; in other cases, doping defective graphene in the coating reduces the interface adhesion work of the model.

**Figure 21:**
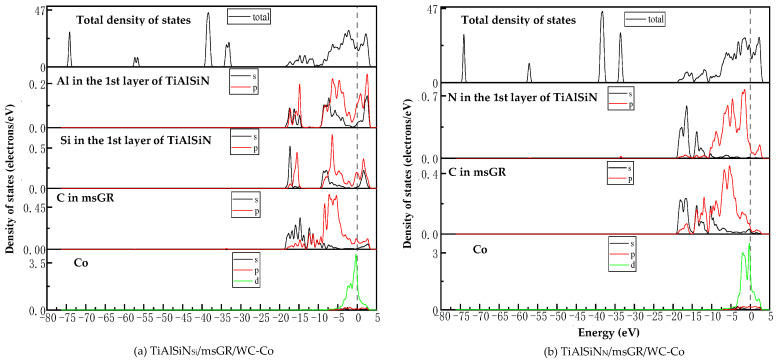
TDOS and PDOS of the interface models with the coating/matrix interface doped with msGR.

Figure 21 shows the images of TDOS and PDOS of the interface models with the coating/matrix interface doped with msGR. As shown in Figure 21a, at the interface of TiAlSiN_Si_/msGR/WC-Co, the density of states of Al and Si atoms overlaps with C atoms of msGR in the range −10~3 eV, and Al-s, Al-p, Si-p, and C-p produce orbital hybridization, forming Al/C and Si/C bonds. The density of states of Co and C atoms overlaps in the range −5~3 eV, and the density of states of C atoms in the overlapping region is very low, the region of density of states of Co is narrow, and the Co/C bond formed by the orbital hybridization of Co-d and C-p is weak, which explains the essential mechanism that the adhesion work of TiAlSiN_Si_/msGR/WC-Co is less than that of TiAlSiN_Si_/WC-Co. In Figure 21b, at the interface of TiAlSiN_N_/msGR/WC-Co, the density of states of N and C atoms of msGR overlaps in the range −20~10 eV, and the N-s and C-s produce orbital hybridization. In the range −10~3 eV, N-p and C-p produce orbital hybridization, which forms strong N/C bonds. The density of states of Co and C atoms overlaps in the range −5~3 eV, and Co-d and C-p produce orbital hybridization, forming strong Co/C bonds, which explains the essential mechanism that the adhesion work of TiAlSiN_N_/msGR/WC-Co is greater than that of TiAlSiN_N_/WC-Co.

**Figure 22:**
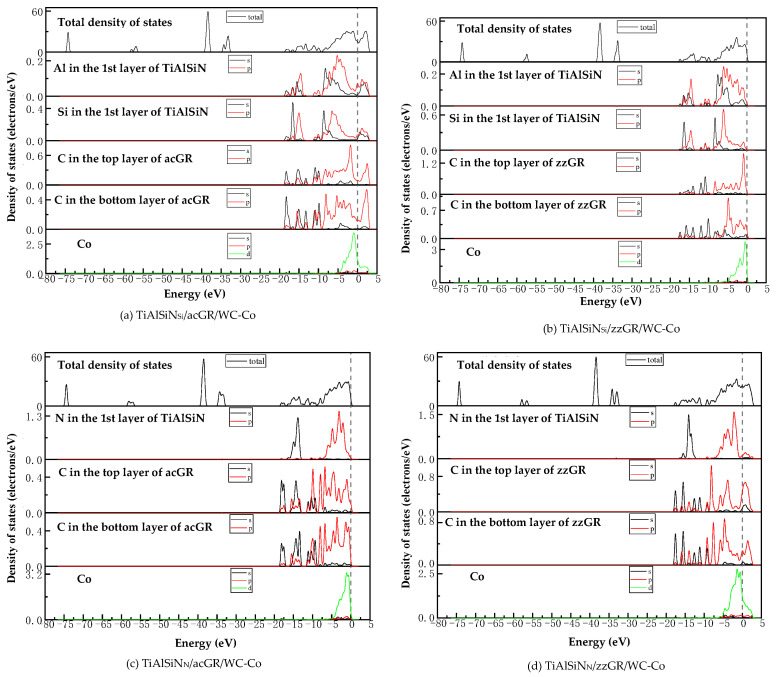
TDOS and PDOS of the interface models with the coating/matrix interface doped with an intrinsic graphene boundary.

Figure 22 shows the images of TDOS and PDOS of the interface models with the coating/matrix interface doped with an intrinsic graphene boundary. As shown in Figure 22a, for the model of TiAlSiN_Si_/acGR/WC-Co, the Al-s, Al-p, Si-s, and C-p produce orbital hybridization in the range −10~3 eV, forming Al/C and Si/C bonds. The Co-d and C-p produce orbital hybridization, and the degree of hybridization is low, forming weak Co/C bonds. In Figure 22b, the density of states of C-p and Co-d in TiAlSiN_Si_/zzGR/WC-Co overlaps in the range −5~0 eV, the C-p density of states is low, and the C/Co bond is weak. In Figure 22c, in the model of TiAlSiN_N_/acGR/WC-Co, the density of states of C-p and N-p overlaps in the range −7~0 eV, the C-p density of states is low, and the N/C bond is weak. In Figure 22d, the density of states of N-p and C-d overlaps in the range −7~0 eV. The degree of orbital hybridization between N-p and C-p is low, and N/C bond is weak. This explains the essential mechanism of the decrease in adhesion work at the TiAlSiN/WC-Co interface when the coating/matrix interface is doped with an intrinsic graphene boundary.

**Figure 23:**
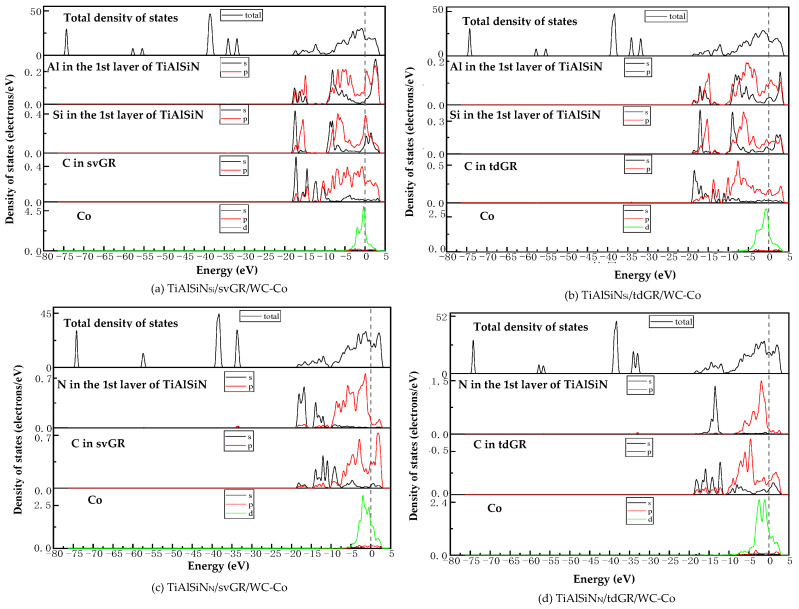
TDOS and PDOS of the interface models with the coating/matrix interface doped with defective graphene.

Figure 23 shows the images of TDOS and PDOS of the interface models with the coating/matrix interface doped with defective graphene. As shown in Figure 23a, in the model of TiAlSiN_Si_/svGR/WC-Co, the density of Al, Si, and C atoms of svGR overlaps in the ranges −17~15 and −10~3 eV. Al-s, Al-p, Si-p, and C-p produce orbital hybridization, forming the Al/C and Si/C bonds. The density of states of Co and C atoms overlaps in the range −4~3 eV. Co-d and C-p produce orbital hybridization, and the degree of hybridization is low, and the Co/C bond is weak. In Figure 23b, in the model of TiAlSiN_Si_/tdGR/WC-Co, the density of Al, Si, and C atoms of svGR overlaps in the ranges −17~15 and −11~3 eV. Al-s, Al-p, Si-s, Si-p, and C-p produce orbital hybridization to form Al/C and Si/C bonds. The density of states of Co and C atoms overlaps in the range −5~4 eV, and Co-d and C-p produce orbital hybridization. The peak value of C-p in the overlapping region is low, and the Co/C bond formed is weak. In Figure 23c, in the model of TiAlSiN_N_/svGR/WC-Co, the density of states of N-s and C-s overlap in the range −15~8 eV. The density of states of N-p and C-p overlap in the range −7~3 eV, and the degree of electron orbital hybridization is high, and the N/C bond is strong. In Figure 23d, N-p, Co-d, and C-p produce orbital hybridization, the degree of orbital hybridization is low, forming weak N/C and Co/C bonds. Therefore, the interface adhesion work of TiAlSiN_N_/svGR/WC-Co is greater than that of TiAlSiN_N_/WC-Co without graphene doping. In other cases, doping defective graphene in the coating/matrix interface reduces the interface adhesion work of the model. The analysis results of density of states are consistent with the analysis results of previous adhesion works, which reveals the interface bonding mechanism of graphene-doped TiAlSiN/WC-Co.

The partial research results are consistent with the experimental results in references [14,21]. Reference [14] results showed that WC-Co doped with GR is prone to produce a bending friction layer. The bending and wrapping of the GR surface are the main toughening mechanisms, which increases the interface bonding strength. In the Co/GR and GR/WC interfaces, the C in GR is prone to distribute the electron clouds closer to W and Co; C-W and C-Co form covalent bonds and the interface bonding is strengthened. Li et al. [21] found that GR could be well attached to WC and around it, thus refining WC grains.

## 4. Conclusions

(1) Based on the most stable and unstable interface models of coated, cemented carbide tools of TiAlSiN_Si_/WC-Co and TiAlSiN_N_/WC-Co, the interface models of graphene-doped matrix, coating, and coating/matrix interface are established, the interface adhesion works are calculated with the first-principles method. The results show that the matrix doped only with msGR improves interface adhesion works of TiAlSiN_Si_/WC/msGR/Co and TiAlSiN_N_/WC/msGR/Co, and the interface bonding property of TiAlSiN/WC-Co improves. The coating doped with msGR and svGR improves adhesion works and interface bonding properties TiAlSiN/WC-Co. Additionally, the coating/matrix interface doped with msGR and svGR improves adhesion works and interface bonding properties of TiAlSiN/WC-Co.

Based on these research results, the electronic structures of the models are further analyzed. The results show that there exists strong Si/Co and N/Co covalent bonds in the interfaces when the matrix is doped with the main surface of intrinsic graphene, which causes the adhesion work of TiAlSiN_Si_/WC/msGR/Co to be greater than that of TiAlSiN_Si_/WC-Co, and the adhesion work of TiAlSiN_N_/WC/msGR/Co to be greater than that of TiAlSiN_N_/WC-Co. Additionally, there exists strong N/Co covalent bonds in the interfaces of TiAlSiN/msGR/TiAlSiN_N_/WC-Co, which lead to the adhesion work of TiAlSiN/msGR/TiAlSiN_N_/WC-Co to be greater than that of TiAlSiN_N_/WC-Co. Additionally, there exists more charge transfer and orbital hybridization in the coating/matrix interface doped with the main surface of intrinsic graphene and single vacancy defective graphene, which leads to the adhesion work of TiAlSiN_N_/msGR/WC-Co to be greater than that of TiAlSiN_N_/WC-Co, and the adhesion work of TiAlSiN_N_/svGR/WC-Co to be greater than that of TiAlSiN_N_/WC-Co.

(2) Electronic structure analysis shows that when the matrix is doped with the main surface of graphene, msGR doping introduces msGR/Co and WC/msGR interfaces. At the interface of TiAlSiN_Si_/WC/msGR/Co, there exists orbital hybridization between the Si p-electron and the Co d-electron, forming strong Si/Co covalent bonds. At the interface of TiAlSiN_N_/WC/msGR/Co, there exists the hybridization of the N p-electron and the Co d-electron, forming stable N/Co covalent bonds. When the matrix is doped with the graphene boundary, C atoms and Co and W atoms produce orbital hybridization, and the hybridization degree and the bonding between Al, Si, N atoms and Co atoms at the interface of TiAlSiN are weak. When the matrix is doped with the defective graphene, the combination of matrix and defective graphene changes the charge distribution at the interface, and the bonding between atoms at the interface of the model is weak.

(3) When the coating is doped with msGR, in the model of TiAlSiN/msGR/TiAlSiN_Si_/WC-Co, there are electronic blank areas between Al, Si, and Co atoms. At the Fermi level, there exists the orbital hybridization between Al, Si atoms, and Co atoms to form weak covalent bonds. At the interface of TiAlSiN/msGR/TiAlSiN_N_/WC-Co, N-p and Co-d produce orbital hybridization to form N/Co covalent bonds. When the coating is doped with the graphene boundary, the charge between atoms at the interface of the model is low, the hybridization degree between atoms at the interface is low, and the bonding is weak. When the coating is doped with the defective graphene, only the atomic charges at the interface of TiAlSiN/svGR/TiAlSiN_N_/WC-Co overlap more, and the degree of hybridization between atoms is high, and the interatomic force is strong. In other cases, the doping defective graphene weakens the bonding force between atoms at the interface.

(4) When the coating/matrix interface is doped with msGR, there exists charge transfer at the interface of TiAlSiN_Si_/msGR/WC-Co, and shared charges between C and Si atoms. The atomic charge density between Co and C atoms is thin, and the Co/C bond is weak, and the interatomic force is weak. At the interface of TiAlSiN_N_/msGR/WC-Co, there are obvious shared charges between C atoms of msGR and N and Co atoms. Co-d and C-p produce orbital hybridization to form strong Co/C bonds, and the interatomic force is strong. When the coating/matrix interface is doped with an intrinsic graphene boundary, the combination of coating/matrix interface and graphene boundary makes the charge between atoms thin, and the orbital hybridization degree between atoms decreases, and the interatomic force is weak. When the coating/matrix interface is doped with the defective graphene, only the atomic charges at the TiAlSiN_N_/svGR/WC-Co interface overlap more, and the orbital hybridization degree between atoms is high and the bonding force is strong. In other interface models, there exists weak electronic orbital hybridization, and the bonding force between atoms is weak. In other cases, doping defective graphene in the coating/matrix interface reduces the interface adhesion work of the models.

## Figures and Tables

**Table 1:** The lattice cell parameters of WC, Co, GR, and TiAlSiN.

Bulk	Lattice Cell Parameters/Å	Experimental Values [34,35,36]/Å
WC	a = b = 2.926, c = 2.849	a = b = 2.906, c = 2.849
Co	a = b = c = 3.544	a = b = c = 3.510
GR	a = b = 2.465, c = 2.574	a = b = 2.460, c = 2.460
TiAlSiN	a = b = 5.991, c = 7.345	a = b = 5.995, c = 7.342

**Table 2:** Adhesion works of matrix doped with intrinsic graphene.

Interface Model	Interface	*E*_α_/eV	*E*_β_/eV	E_α/β_/eV	*A*_α/β_/Å^2^	*W*_ad_/J·m^−2^
TiAlSiN_Si_/WC-Co	TiAlSiN_Si_/Co	−8864.548	−19,214.219	−28,096.425	29.688	9.517
TiAlSiN_Si_/WC/msGR/Co	TiAlSiN_Si_/Co	−8857.649	−20,351.550	−29,228.776	25.510	12.279
	msGR/Co	−11,900.242	−17,305.430	−29,228.776	25.510	14.491
	WC/msGR	−13,135.583	−16,070.392	−29,228.776	25.510	14.301
TiAlSiN_Si_/WC/acGR/Co	TiAlSiN_Si_/Co	−8857.077	−21,075.123	−29,934.821	25.510	1.644
	Co/acGR	−12,003.198	−17,912.225	−29,934.821	25.510	12.167
	acGR/WC	−13,823.387	−16,090.325	−29,934.821	25.510	13.240
TiAlSiN_Si_/WC/zzGR/Co	TiAlSiN_Si/_Co	−8858.354	−20,691.385	−29,552.824	25.510	1.935
	Co/zzGR	−11,938.665	−17,599.367	−29,552.824	25.510	9.278
	zzGR/WC	−13,516.226	−16,024.339	−29,552.824	25.510	7.689
TiAlSiN_N_/WC-Co	TiAlSiN_N_/Co	−8869.501	−19,213.712	−28,089.436	30.401	3.280
TiAlSiN_N_/WC/msGR/Co	TiAlSiN_N_/Co	−8856.045	−20,387.355	−29,253.555	25.510	6.369
	msGR/Co	−11,982.997	−17,250.534	−29,253.555	25.510	12.559
	WC/msGR	−13,219.437	−16,016.384	−29,253.555	25.510	11.123
TiAlSiN_N_/WC/acGR/Co	TiAlSiN_N_/Co	−8858.972	−20,976.595	−29,840.519	28.841	2.747
	Co/acGR	−11,983.842	−17,840.524	−29,840.519	28.841	8.961
	acGR/WC	−13,822.552	−16,005.100	−29,840.519	28.841	7.138
TiAlSiN_N_/WC/zzGR/Co	TiAlSiN_N_/Co	−8845.812	−20,705.731	−29,554.548	25.510	1.885
	Co/zzGR	−11,966.275	−17,579.687	−29,554.548	25.510	5.385
	zzGR/WC	−13,492.722	−16,051.485	−29,554.548	25.510	6.486

**Table 3:** Adhesion works of matrix doped with defective graphene.

Interface Model	Interface	*E*_α_/eV	*E*_β_/eV	E_α/β_/eV	*A*_α/β_/Å^2^	*W*_ad_/J·m^−2^
TiAlSiN_Si_/WC/svGR/Co	TiAlSiN_Si_/Co	−8985.802	−20,105.445	−29,096.815	25.510	3.492
	Co/svGR	−12,960.125	−16,129.989	−29,096.815	25.510	4.203
	svGR/WC	−13,219.437	−15,859.432	−29,096.815	25.510	11.256
TiAlSiN_Si_/WC/tdGR/Co	TiAlSiN_Si_/Co	−8849.947	−20,388.517	−29,251.544	30.401	6.884
	Co/tdGR	−11,965.212	−17,277.746	−29,251.544	30.401	4.519
	tdGR/WC	−13,194.841	−16,039.931	−29,251.544	30.401	8.827
TiAlSiN_N_/WC/svGR/Co	TiAlSiN_N_/Co	−8844.718	−20,266.608	−29,114.923	25.510	2.256
	Co/svGR	−11,965.966	−17,145.177	−29,114.923	25.510	2.371
	svGR/WC	−13,040.8	−16,063.819	−29,114.923	25.510	6.463
TiAlSiN_N_/WC/tdGR/Co	TiAlSiN_N_/Co	−8923.718	−20,343.056	−29,269.889	18.373	2.713
	Co/tdGR	−12,965.966	−16,301.796	−29,269.889	18.373	1.852
	tdGR/WC	−13,240.821	−16,019.288	−29,269.889	18.373	8.517

**Table 4:** Adhesion works of coating doped with intrinsic graphene.

Interface Model	Interface	*E*_α_/eV	*E*_β_/eV	E_α/β_/eV	*A*_α/β_/Å^2^	*W*_ad_/J·m^−2^
TiAlSiN_Si_/WC-Co	TiAlSiN_Si_/Co	−8864.548	−19,214.219	−28,096.425	29.688	9.517
TiAlSiN/msGR/TiAlSiN_Si_/WC-Co	TiAlSiN/msGR	−4399.354	−24,796.497	−29,212.198	25.510	10.253
	msGR/TiAlSiN_Si_	−5632.754	−23,560.592	−29,212.198	25.510	11.824
	TiAlSiN_Si_/Co	−10,070.644	−19,133.718	−29,212.198	25.510	4.915
TiAlSiN/acGR/TiAlSiN_Si_/WC-Co	TiAlSiN/acGR	−4397.399	−25,416.425	−29,825.798	25.510	7.51
	acGR/TiAlSiN_Si_	−6238.117	−23,585.309	−29,825.798	25.510	1.488
	TiAlSiN_Si_/Co	−10,686.429	−19,136.934	−29,825.798	25.510	1.527
TiAlSiN/zzGR/TiAlSiN_Si_/WC-Co	TiAlSiN/zzGR	−4397.97	−25,108.831	−29,522.271	25.510	9.703
	zzGR/TiAlSiN_Si_	−5926.168	−23,586.173	−29,522.271	25.510	6.228
	TiAlSiN_Si_/Co	−10,381.551	−19,138.976	−29,522.271	25.510	1.094
TiAlSiN_N_/WC-Co	TiAlSiN_N_/Co	−8869.501	−19,213.712	−28,089.436	30.401	3.280
TiAlSiN/msGR/TiAlSiN_N_/WC-Co	TiAlSiN/msGR	−4539.354	−24,646.868	−29,206.163	25.510	12.507
	msGR/TiAlSiN_N_	−5632.554	−23,561.909	−29,206.163	25.510	7.338
	TiAlSiN_N_/Co	−10,170.23	−19,026.266	−29,206.163	25.510	6.063
TiAlSiN/acGR/TiAlSiN_N_/WC-Co	TiAlSiN/acGR	−4439.424	−25,392.146	−29,835.169	30.401	1.894
	acGR/TiAlSiN_N_	−5732.521	−24,091.894	−29,835.169	30.401	5.66
	TiAlSiN_N_/Co	−10,087.232	−19,736.696	−29,835.169	30.401	5.916
TiAlSiN/zzGR/TiAlSiN_N_/WC-Co	TiAlSiN/zzGR	−4447.144	−25,070.642	−29,522.22	25.510	2.781
	zzGR/TiAlSiN_N_	−5977.433	−23,539.290	−29,522.22	25.510	3.448
	TiAlSiN_N_/Co	−10,380.04	−19,133.030	−29,522.22	25.510	5.739

**Table 5:** Adhesion works of coating doped with defective graphene.

Interface Model	Interface	*E*_α_/eV	*E*_β_/eV	E_α/β_/eV	*A*_α/β_/Å^2^	*W*_ad_/J·m^−2^
TiAlSiN/svGR/TiAlSiN_Si_/WC-Co	TiAlSiN/svGR	−4398.545	−24,653.654	−29,063.113	20.510	8.514
	svGR/TiAlSiN_Si_	−5475.526	−23,573.913	−29,063.113	20.510	10.667
	TiAlSiN_Si_/Co	−9921.346	−19,139.966	−29,063.113	20.510	1.405
TiAlSiN/tdGR/TiAlSiN_Si_/WC-Co	TiAlSiN/tdGR	−4403.932	−24,795.696	−29,221.646	30.401	11.588
	tdGR/TiAlSiN_Si_	−5629.558	−23,588.449	−29,221.646	30.401	1.915
	TiAlSiN_Si_/Co	−10,078.037	−19,136.894	−29,221.646	30.401	3.534
TiAlSiN/svGR/TiAlSiN_N_/WC-Co	TiAlSiN/svGR	−4448.474	−24,602.942	−29,061.557	20.510	7.911
	svGR/TiAlSiN_N_	−5517.203	−23,527.760	−29,061.557	20.510	12.945
	TiAlSiN_N_/Co	−9918.363	−19,137.165	−29,061.557	20.510	4.703
TiAlSiN/tdGR/TiAlSiN_N_/WC-Co	TiAlSiN/tdGR	−4452.335	−24,751.771	−29,218.480	30.401	7.565
	tdGR/TiAlSiN_N_	−5669.858	−23,543.024	−29,218.480	30.401	1.852
	TiAlSiN_N_/WC-Co	−10,077.266	−19,137.520	−29,218.480	30.401	8.517

**Table 6:** Adhesion works of coating/matrix interface doped with intrinsic graphene.

Interface Model	Interface	*E*_α_/eV	*E*_β_/eV	E_α/β_/eV	*A*_α/β_/Å^2^	*W*_ad_/J·m^−2^
TiAlSiN_Si_/WC-Co	TiAlSiN_Si_/Co	−8864.548	−19,214.219	−28,096.425	29.688	9.517
TiAlSiN_Si_/msGR/WC-Co	TiAlSiN_Si_/GR	−8862.227	−20,418.426	−29,289.112	25.510	5.305
	GR/WC-Co	−9940.304	−19,344.691	−29,289.112	25.510	2.582
TiAlSiN_Si_/acGR/WC-Co	TiAlSiN_Si_/acGR	−8862.227	−20,418.426	−29,289.112	25.510	8.475
	acGR/WC-Co	−9940.304	−19,344.691	−29,289.112	25.510	5.264
TiAlSiN_Si_/zzGR/WC-Co	TiAlSiN_Si_/zzGR	−8853.822	−20,261.822	−29,129.156	25.510	9.212
	zzGR/WC-Co	−9953.141	−19,167.622	−29,129.156	25.510	2.088
TiAlSiN_N_/WC-Co	TiAlSiN_N_/Co	−8869.501	−19,213.712	−28,089.436	30.401	3.280
TiAlSiN_N_/msGR/WC-Co	TiAlSiN_N_/GR	−8858.828	−20,288.544	−29,160.792	25.510	8.417
	GR/WC-Co	−9933.179	−19,217.321	−29,160.792	25.510	6.455
TiAlSiN_N_/acGR/WC-Co	TiAlSiN_N_/acGR	−8858.828	−20,288.544	−29,160.792	25.510	1.257
	acGR/WC-Co	−9933.179	−19,217.321	−29,160.792	25.510	5.771
TiAlSiN_N_/zzGR/WC-Co	TiAlSiN_N_/zzGR	−8858.828	−20,319.338	−29,180.554	30.401	2.452
	zzGR/WC-Co	−9933.179	−19,236.410	−29,180.554	30.401	5.090

**Table 7:** Adhesion works of coating/matrix interface doped with defective graphene.

Interface Model	Interface	*E*_α_/eV	*E*_β_/eV	E_α/β_/eV	*A*_α/β_/Å^2^	*W*_ad_/J·m^−2^
TiAlSiN_Si_/svGR/WC-Co	TiAlSiN_Si_/svGR	−8862.227	−20,289.709	−29,162.669	25.510	6.732
	svGR/WC-Co	−9940.304	−19,213.382	−29,162.669	25.510	5.634
TiAlSiN_Si_/tdGR/WC-Co	TiAlSiN_Si_/tdGR	−8863.677	−20,444.369	−29,316.361	30.401	4.376
	tdGR/WC-Co	−10,093.936	−19,214.859	−29,316.361	30.401	3.982
TiAlSiN_N_/svGR/WC-Co	TiAlSiN_N_/svGR	−8858.828	−20,288.546	−29,160.792	25.510	8.416
	svGR/WC-Co	−9933.179	−19,219.523	−29,160.792	25.510	5.074
TiAlSiN_Si_/tdGR/WC-Co	TiAlSiN_N_/tdGR	−8861.527	−20,444.409	−29,311.288	30.401	2.817
	tdGR/WC-Co	−10,089.876	−19,217.340	−29,311.288	30.401	2.143

**Table 8:** Adhesion works of TiAlSiN/WC-Co interface doped with graphene.

			Graphene	Intrinsic Graphene			Defective Graphene	
	Adhesion			Main Surface of Graphene	Graphene Boundary			
Doping Position				msGR	acGR	zzGR	svGR	tdGR
WC-Co matrix		Si terminal		12.279	1.644	1.935	3.492	4.519
		N terminal		6.369	2.747	1.885	2.256	1.852
TiAlSiN coating		Si terminal		4.915	1.488	1.094	1.405	1.915
		N terminal		6.063	1.894	2.781	4.703	1.944
Coating/matrix interface		Si terminal		2.592	5.264	2.088	5.634	3.982
		N terminal		6.455	1.257	2.452	5.074	2.143

## Data Availability

Data sharing not applicable.

## Supplementary Materials

The following supporting information can be downloaded at: https://www.mdpi.com/article/10.3390/mi14020431/s1, Figure S1: Interface models of matrix doped with defective graphene: (a) TiAlSiN_Si_/WC/svGR/Co; (b) TiAlSiN_Si_/WC/tdGR/Co; (c) TiAlSiN_N_/WC/svGR/Co; (d) TiAlSiN_N_/WC/tdGR/Co.; Figure S2: Interface models of coating doped with intrinsic graphene: (a) TiAlSiN/msGR/TiAlSiN_Si_/WC-Co; (b) TiAlSiN/acGR/TiAlSiN_Si_/WC-Co; (c) TiAlSiN/zzGR/TiAlSiN_Si_/WC-Co; (d) TiAlSiN/msGR/TiAlSiN_N_/WC-Co; (e) TiAlSiN/acGR/TiAlSiN_N_/WC-Co; (f) TiAlSiN/zzGR/TiAlSiN_N_/WC-Co.; Figure S3: Interface models of coating doped with defective graphene: (a) TiAlSiN/svGR/TiAlSiN_Si_/WC-Co; (b) TiAlSiN/tdGR/TiAlSiN_Si_/WC-Co; (c) TiAlSiN/svGR/TiAlSiN_N_/WC-Co; (d) TiAlSiN/tdGR/TiAlSiN_N_/WC-Co. Figure S4: Interface models of coating/matrix interface doped with intrinsic graphene: (a) TiAlSiN_Si_/msGR/WC-Co; (b) TiAlSiN_Si_/acGR/WC-Co;. (c) TiAlSiN_Si_/zzGR/WC-Co; (d) TiAlSiN_N_/msGR/WC-Co; (e) TiAlSiN_N_/acGR/WC-Co; (f) TiAlSiN_N_/zzGR/WC-Co; Figure S5: Interface models of coating/matrix interface doped with defective graphene: (a) TiAlSiN_Si_/svGR/WC-Co; (b) TiAlSiN_Si_/tdGR/WC-Co; (c) TiAlSiN_N_/svGR/WC-Co; (d) TiAlSiN_N_/tdGR/WC-Co.
