# Supplementary material for: Research on Interface Bonding Properties of TiAlSiN/WC-Co Doped with Graphene
Source: Micromachines (Basel). 2023 Feb 11;14(2):431. doi: 10.3390/mi14020431 (PMC9966344; doi:10.3390/mi14020431)
Supplement: Supplementary file 1 [file micromachines-14-00431-s001.zip › micromachines-2161105-supplementary.pdf]

micromachines

MDPI

Article

Research on Interface Bonding Properties of TiAlSiN/WC-Co Doped with Graphene

Junru Yang *, Yan Wang, Hao Lv, Yanping Yue, Shulei Li and Ran Zhu

College of Mechanical and Electronic Engineering, Shandong University of Science and Technology, Qingdao 266590, China

* Correspondence: yangjunru@sdust.edu.cn

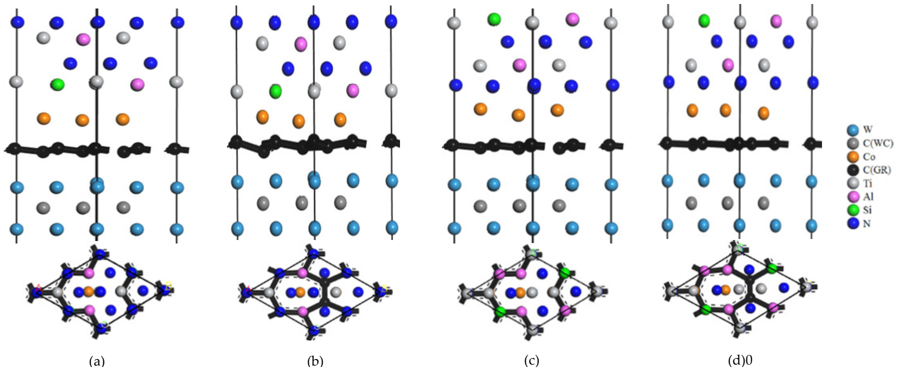

Figure S1. Interface models of matrix doped with defective graphene: (a) $TiAlSiN_{Si}$/WC/svGR/Co; (b) $TiAlSiN_{Si}$/WC/tdGR/Co; (c) $TiAlSiN_{N}$/WC/svGR/Co; (d) $TiAlSiN_{N}$/WC/tdGR/Co.

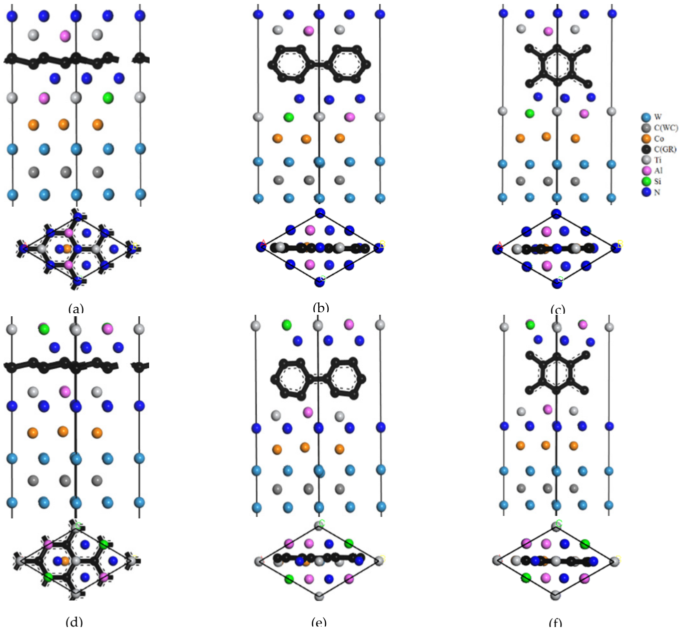

Figure S2. Interface models of coating doped with intrinsic graphene: (a) TiAlSiN/msGR/TiAlSiN$_{Si}$/WC-Co; (b) TiAlSiN/acGR/TiAlSiN$_{Si}$/WC-Co; (c) TiAlSiN/zzGR/TiAlSiN$_{Si}$/WC-Co; (d) TiAlSiN/msGR/TiAlSiN$_{N}$/WC-Co; (e) TiAlSiN/acGR/TiAlSiN$_{N}$/WC-Co; (f) TiAlSiN/zzGR/TiAlSiN$_{N}$/WC-Co.

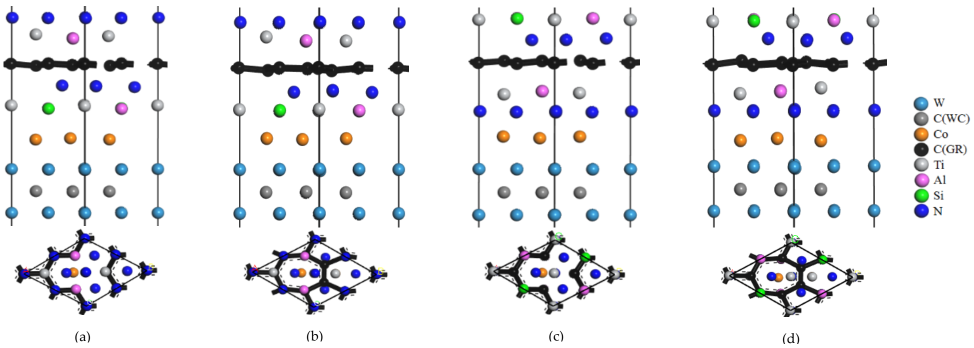

Figure S3. Interface models of coating doped with defective graphene: (a) TiAlSiN/svGR/TiAlSiN$_{Si}$/WC-Co; (b) TiAlSiN/tdGR/TiAlSiN$_{Si}$/WC-Co; (c) TiAlSiN/svGR/TiAlSiN$_{N}$/WC-Co; (d) TiAlSiN/tdGR/TiAlSiN$_{N}$/WC-Co.

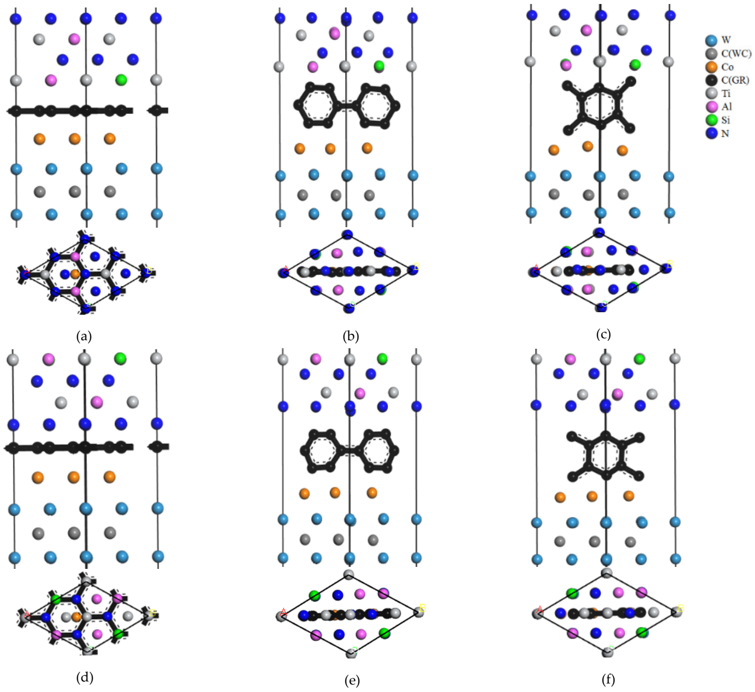

Figure S4. Interface models of coating/matrix interface doped with intrinsic graphene: (a) TiAlSiN$_{Si}$/msGR/WC-Co; (b) TiAlSiN$_{Si}$/acGR/WC-Co;. (c) TiAlSiN$_{Si}$/zzGR/WC-Co; (d) TiAlSiN$_{N}$/msGR/WC-Co; (e) TiAlSiN$_{N}$/acGR/WC-Co; (f) TiAlSiN$_{N}$/zzGR/WC-Co.

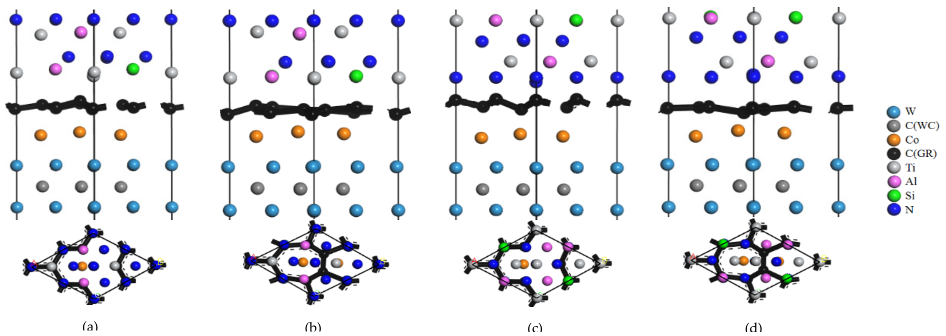

Figure S5. Interface models of coating/matrix interface doped with defective graphene: (a) $TiAlSiN_{Si}$/svGR/WC-Co; (b) $TiAlSiN_{Si}$/tdGR/WC-Co; (c) $TiAlSiN_N$/svGR/WC-Co; (d) $TiAlSiN_N$/tdGR/WC-Co.
